# Applications of Plant Protein in the Dairy Industry

**DOI:** 10.3390/foods11081067

**Published:** 2022-04-07

**Authors:** Ge-Ge Hu, Jing Liu, Yi-Hui Wang, Zhen-Nai Yang, Hong-Bo Shao

**Affiliations:** 1 Beijing Advanced Innovation Center for Food Nutrition and Human Health, Beijing Engineering and Technology Research Center of Food Additives, Beijing Technology and Business University, No. 11 Fu-Cheng Road, Hai-Dian District, Beijing 100048, China; 2050201003@st.btbu.edu.cn (G.-G.H.); 2030201010@st.btbu.edu.cn (J.L.); 2030201018@st.btbu.edu.cn (Y.-H.W.); 2 Jiangsu Key Laboratory for Bioresources of Saline Soils, Jiangsu Synthetic Innovation Center for Coastal Bio-Agriculture, Yancheng Teachers University, Yancheng 224002, China; 3 Salt-Soil Agricultural Center, Institute of Agricultural Resources and Environment, Jiangsu Academy of Agriculture Sciences (JAAS), Nanjing 210014, China

**Keywords:** plant protein, double protein dairy, process flow, production, health effects, taste, flavor

## Abstract

In recent years, a variety of double protein dairy products have appeared on the market. It is a dairy product made by replacing parts of animal protein with plant protein and then using certain production methods. For some countries with limited milk resources, insufficient protein intake and low income, double protein dairy products have a bright future. More and more studies have found that double protein dairy products have combined effects which can alleviate the relatively poor functional properties of plant protein, including solubility, foaming, emulsifying and gelling. In addition, the taste of plant protein has been improved. This review focuses on the current state of research on double protein dairy products. It covers some salient features in the science and technology of plant proteins and suggests strategies for improving their use in various food applications. At the same time, it is expected that the fermentation methods used for those traditional dairy products as well as other processing technologies could be applied to produce novelty foods based on plant proteins.

## 1. Introduction

Protein is the material basis of all life and plays a very important role in maintaining the normal growth, metabolism and immune regulation of the body. According to the source of intake, protein can be divided into animal protein and plant protein. Animal protein is rich in a lot of essential amino acids, but excessive intake of animal protein will greatly increase fat intake. Saturated fatty acids as the main component will lead to increased plasma cholesterol levels, which is not conducive to human health [1]. Due to the improvement of people’s living standards and the gradual increase in protein intake, the supply of animal protein is not enough to meet people’s growing demand [2]. Plant protein has the advantages of abundant resources, being cheap and easy to obtain, having no cholesterol, and it can prevent diseases [3]. It is a green and safe food raw material, which can make up for the insufficient supply of animal protein.

In 2006, the “Shanghai Declaration” was released at the “Second China Soybean Food Industry Roundtable Summit” held in Shanghai, which was the first time that China proposed the concept of “double protein”. The concept of “double protein” and the “double protein” strategy were put forward for the first time, emphasizing the combination of soy protein and milk protein to meet the health needs of comprehensive protein supplementation. It is pointed out that efforts should be made to develop new nutritional and healthy foods such as nutritionally fortified foods and double protein foods. With people’s further awareness of nutrition and health, high-nutrition and high-protein products are more and more attractive to consumers [4].

Dairy products are liquid or solid products made from milk as the main raw material through heating, drying and fermentation processes. Dairy products are rich in protein, fat and carbohydrates, which are easy to digest and absorb. In particular, it contains vitamins and calcium, which are an excellent source of nutrients needed by the human body. As a nutritious and comprehensive ideal food, dairy products occupy a very important position in the dietary structure [5]. Due to the existence of healthy long-chain unsaturated fatty acids, the development of dairy products based on plant protein endows dairy products with new nutrition and health care connotations. It not only realizes the functionalization and popularization of dairy products, but also promotes the diversified development of the dairy processing industry.

## 2. Double Protein Dairy

### 2.1. Double Protein Yogurt

Yogurt is a fast acid-producing product, which is made from raw cow (goat) milk or milk powder after high-speed homogenization, sterilization and fermentation. Due to its unique flavor and texture, it is currently the most widely distributed and consumer favorite fermented dairy product in the world. Double protein yogurt uses plant protein and animal protein as the main nutritional bases. Through probiotic fermentation, it has a unique flavor and high nutritional value, which helps to improve nutrition and improve human health.

During the fermentation process of yogurt, the performance of the starter greatly affects the quality of yogurt. Therefore, the development of probiotic strains with excellent fermentation performance is the key to the preparation of a highly active starter [6]. At present, dairy production enterprises mainly use *Streptococcus thermophilus* and *Lactobacillus bulgaricus* as starters for producing yogurt. In recent years, research on plant protein yogurt starter has been increasing. In the fermentation of suitable strains of plant-based yogurt, one or more functional strain combinations can be included.

Sertovic et al. [7] mixed *Streptococcus thermophilus*, *Lactobacillus bulgaricus* and *Lactobacillus acidophilus* to ferment milk and soymilk, and they found that the acidity of fermented milk was higher than that of fermented soymilk. This showed that the optimal starter for soy protein yogurt was slightly different from that of ordinary milk yogurt. Havas et al. [8] fermented pure soymilk using human-derived bacteria *Bifidobacterium* B3.2 and *Bifidobacterium* B7.1. The results showed that the acid-producing ability of the two kinds of bifidobacteria in soymilk was similar to that of cow’s milk, and there was no unpleasant odor. Li et al. [9] found that both *Lactobacillus plantarum* fs-4 and *Lactobacillus casei* 05-20 had protease activity. They could utilize nutrients such as sugar and protein in peanut milk and were suitable for fermenting peanut milk. The obtained peanut yogurt was white in color and had a peanut and milk flavor. Wan et al. [10] used rice-milk double-protein yogurt fermented by *Lactobacillus furfur* or *Lactococcus lactis* subsp. *lactis*. When the degree of hydrolysis was 7.5%, the sensory score was the highest, which showed that the color was bright, the curd state was stable, the taste was fine and there was no bad flavor. When the degree of hydrolysis exceeded 7.5%, the bitterness of the product became heavier. The main reason was that the rice protein was hydrolyzed to a certain extent and produces bitter peptides. Wang [11] found that mixed strain fermentation had a better effect on the overall quality of coconut yogurt than single strain fermentation. When the addition amount of coconut milk was 30% and the addition amount of whey protein and sodium caseinate was 2%, the starter was mixed and fermented with *Streptococcus thermophilus*, *Lactobacillus bulgaricus*, *Lactobacillus casei* and *Lactobacillus plantarum* in the same proportion. Coconut yogurt was the best quality.

**Figure 1:**
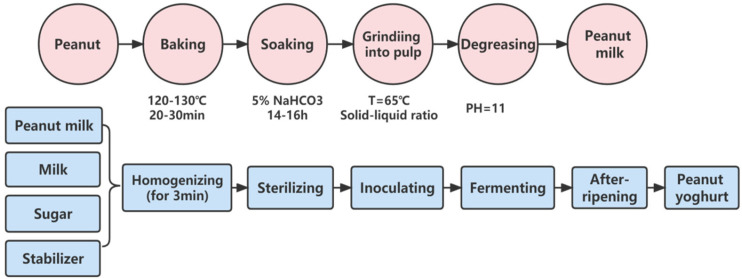
Process flow chart showing some common methods and steps used for production of peanut protein and peanut yogurt.

Taking peanut yogurt as an example, peanuts are rich in protein and essential amino acids, and the nutritional composition of peanut protein and animal protein is similar. If the peanut milk is simply fermented with lactic acid bacteria, the fat content in the peanut kernel is too high, which will lead to oil circles. The high fiber content in peanuts will also affect the formation of peanut yogurt gel. Therefore, milk powder can be added to the peanut yoghurt, so that the peanut yoghurt gel can be formed stably. Peanut yogurt is made of peanuts and milk or milk powder as the main raw materials. After lactic acid bacteria fermentation, it has the characteristics of complementary animal and plant protein and reasonable nutritional structure [12]. The process flow of peanut milk and peanut yogurt is shown in (Figure 1). The researchers studied the ratio of peanut protein and milk or milk powder and obtained peanut yogurt with unique flavor and good taste.

Qin et al. [13] reported the best production process parameters for peanut protein yogurt: the ratio of peanut protein powder to water was 1:9.4, pure milk 15%, whey protein powder 2%, white sugar 10%, starter 3% and fermentation time 7.4 h. The obtained sensory score was 38.02% higher than that before the previous optimization. Compared with the conventional nutritional content of commercially available yogurt, peanut protein yogurt was found to have at least 81.07% higher protein content, at least 25.08% lower fat content, and no significant difference in acidity. According to the acidity, taste and product status, Yang et al. [14] found that the optimal inoculum of lactic acid bacteria-fermented peanuts was about 3%, and the mass ratio of peanut milk, white granulated sugar and milk powder was 90:5:3.5. The obtained peanut yogurt had both the rich aroma of peanut and the flavor of yogurt, and the curd was dense and shiny. Tong [15] found that the ratio of peanut protein to milk protein was 1:2, and then added 0.02% konjac flour and 0.1% soybean polysaccharide for compounding. The peanut yogurt was glossy, evenly curd, moderately sweet and sour, and had a peanut flavor. Ma et al. [16] found that the optimal formula for sprouted peanut yogurt was 8% sugar and 2% sprouted peanut freeze-dried powder. Sprouted peanut yogurt had higher protein content than regular yogurt and slightly lower moisture content. Fang et al. [17] selected *Lactobacillus Delbruckii* as the strain for fermentation, and used purple potato, peanut milk and milk powder as the main raw materials. The peanut and purple potato were organically fermented, and the nutrients of the two were retained. The prepared purple potato peanut yogurt had a mellow taste, full color and greatly improved antioxidant activity. Cao et al. [18] used shelled ginkgo nuts and peanuts as the main raw materials to determine the optimal formula of ginkgo peanut yogurt. The mass ratio of ginkgo to peanut was 1:6, the volume ratio of peanut milk to milk was 6:4, the added sugar was 7% and the inoculum of *Bacillus bulgaricus* and *Streptococcus thermophilus* was 3%. It was possible to obtain nutritious yogurt with a unique taste and delicate organization.

At present, in addition to the scientific research and manufacturing of double-protein yogurt with peanuts as raw materials, double-protein yogurt with beans, nuts, cereals and other plants as raw materials are also emerging one after another. Bruckner-Guhmann et al. [19] reported that the gel strength and elasticity of fermented milk added with oat protein were lower than those of pure fermented milk. However, the sensory evaluation results showed that the fermented milk containing oat protein had better taste, more delicate texture, and had a significant oat flavor. Su et al. [20] mixed pecan milk and milk to develop pecan milk yogurt. Pecan milk yogurt had higher antioxidant properties than regular yogurt. Total solids, fat, crude protein, and amino acids were also higher. Appearance and flavor scores were not significantly different from regular yogurt, but texture scores were higher. Li et al. [21] developed coagulated pea yogurt with pea protein powder and milk as the main raw materials. When the added amount of pea protein powder was 6%, the obtained yogurt had moderate acidity and good quality characteristics. The sensory evaluation was the highest, with a mixed aroma of pea protein powder and yogurt. Chang et al. [22] fermented quinoa milk with 5% puffed quinoa flour and milk as raw materials. At this time, the consistency and acidity of quinoa yogurt had reached the optimal level. Compared with ordinary yogurt, 30 new flavor substances were detected in quinoa yogurt, including six plant-derived terpene compounds and alpha-terpineol. Therefore, the addition of puffed quinoa powder made yogurt have certain advantages in terms of nutrition and flavor. Yang et al. [23] found that the fat content of quinoa yogurt was much lower than that of ordinary yogurt, but the protein content was higher than that of ordinary yogurt. At the same time, it was rich in 8 essential amino acids, among which the content of essential amino acids such as isoleucine and leucine were significantly higher than other yogurt. Gao et al. [24] found that adding about 0.2% soybean and corn combination peptide and 87.5% trehalose to milk powder, the obtained soybean and corn combination peptide fermented milk had the best quality. The smell was refreshing, the taste was delicate, the ingredients were uniform and it had the nutritional and health care functions of soybean polypeptide and rice polypeptide.

Therefore, combining the advantages of plant protein and yogurt can not only enrich the product variety of yogurt products, but also more in line with contemporary people’s pursuit of health and nutrition.

### 2.2. Double Protein Beverages

With the development of science and technology, people’s pursuit of health is getting higher and higher. The development of beverages has entered a new stage, from the original scale growth to the quality upgrade. As a result, the market share of carbonated beverages has continued to decline, and the consumption trend of healthy and natural beverages such as plant protein beverages, fruit and vegetable juices and tea beverages has risen.

Today, plant-based cereal and nut beverages are the newcomers to the dairy industry, but there are some technical challenges in making new dairy products from cereals and nuts. Compared with natural milk, some grains and nuts are rich in starch and fiber. The suspension stability in milk beverages is poor, so the phenomenon of particle suspension and stratification precipitation is easy to occur. In addition, the taste of beverage products is light and bitter. In order to solve these problems, researchers have conducted related research in recent years, including adding stabilizers and thickeners to stabilize product quality; adding flavor substances to improve taste; enriching and strengthening nutrients to improve nutritional value, etc.

Yang et al. [25] studied the compounding scheme of emulsion stabilizer in oat milk beverage. Orthogonal test results showed that the compound stabilizer ingredients included 0.3% microcrystalline cellulose, 0.012% carrageenan, 0.10% mono- and diglyceride fatty acid esters and 0.06% sodium stearoyl lactylate. The stabilizer could effectively suspend product particles and had a good effect of controlling product fat floating. Li et al. [26] determined that the optimal ratio of peanut pulp and milk was 1:2, the stabilizers were sucrose fatty acid ester (SE) 0.05%, glycerol monostearate (GMS) 0.1%, carboxymethyl cellulose sodium (CMC-Na) 0.025%. The produced beverage had good stability. Han et al. [27] found that the addition of walnut juice was 15%, the addition of peanut juice was 25%, the addition of milk was 30%, and the addition of sucrose was 6%. The developed compound milk beverage had the best taste flavor. Under the optimal process conditions, when sodium alginate, gum arabic and CMC were selected as stabilizers, the precipitation rate of the composite beverage decreased and its stability was the best. Huang et al. [28] added 0.04% pectin, 0.02% gellan gum and 0.6% CMC in the production of fermented walnut milk beverages, which not only solved the problem of layered precipitation, but also gave the beverage rich taste.

Enzymatic hydrolysis is the use of amylase or protease to enzymatically hydrolyze macromolecular substances such as starch and protein in grains or nuts under certain conditions. This will refine the granules in the drink, decompose some insoluble starch and protein into soluble sugar, dextrin, polypeptide and amino acid, thereby improving the stability of the drink [29]. Hou et al. [30] used oat as the main raw material and added 0.15% α-amylase for enzymatic hydrolysis. Then, added 1.50% whole milk powder, 3.0% white sugar, 0.15% citric acid, 0.10% pectin and 0.05% xanthan gum to develop the best production process of a new type of cereal beverage. Li et al. [31] used walnut pulp and pea milk as the main raw materials, the addition of milk was 10%, and the addition of white sugar was 3%. The amount of α-amylase added was 0.4%, and the enzymatic hydrolysis was carried out at 70 °C for 3 h. The walnut and pea milk produced under this condition was stable and had the aroma of walnut kernels and peas.

In addition, homogenization can make fat globules smaller. The miniaturization and homogenization of suspended particles can prevent the separation of finished fat and the precipitation of protein particles, thereby improving the emulsification and stability of liquid grain dairy products. Two important parameters of homogenization are homogenization pressure and homogenization temperature. Ma et al. [32] determined the optimal homogenization conditions in the stability study of black glutinous rice milk beverage, that is, homogenized twice under the conditions of 60 °C and 20–30 MPa. When the homogenization temperature was too high, the protein in the system might denature and cause flocculation. When the homogenization pressure exceeded 40 MPa, the number of collisions of suspended particles in the system increased, resulting in polymerization, which eventually led to an increase in the precipitation rate of the system.

From the perspective of raw materials, the taste of beverages can be improved by adding natural raw materials. Zheng et al. [33] used barley and buckwheat as raw materials. Passion fruit juice (10%), xylitol (10%) and citric acid (0.05%) were added to enhance the taste, and the resulting compound grain beverage was rich in aroma and sweet in taste. Wang et al. [34] used walnut juice and milk as the main raw materials. With the addition of 10% macadamia juice, the drink tasted best and had a special aroma of walnuts and macadamia nuts. Zhang et al. [35] used peanut, wolfberry and milk as raw materials, white granulated sugar and xanthan gum as ingredients to develop peanut and wolfberry milk. The study found that the best roasting temperature for peanuts was 120 °C, and the best roasting time was 20 min, the peanuts had the strongest aroma.

From the perspective of preparation, suitable flavor substances can be derived by means of fermentation. Tavares et al. [36] pointed out that organic acids such as lactic acid and acetic acid are released during fermentation and refrigeration of corn beverages fermented with probiotics and yeast. Maintaining the pH of beverages at around 4.0 had an important impact on food safety, taste and aroma. Tue et al. [37] dried and ground germinated brown rice into powder, and fermented after adding honey, corn germ oil and yeast. After adding milk, white sugar and citric acid, it was homogenized to make a brown rice enzyme milk drink with unique flavor.

The double protein beverage has rich raw material resources, meets the individual needs of consumers, conforms to the development trend of market consumption and has a certain health care value, so it has broad development prospects.

### 2.3. Double Protein Cheese

Cheese is made from cow or goat milk. Adding an appropriate amount of starter and rennet can make the protein coagulate, discharge part of the whey, and finally ferment and mature after a certain period of time. During the stage of cheese fermentation, proteins and fats are enzymatically decomposed into tiny substances that are easily absorbed in the human digestive system, which improves the absorption and utilization rate of cheese. Therefore, it has the reputation of milk gold in the industry.

In recent years, a mixed cheese has appeared on the market, which is a cheese made by replacing part of the protein in animal milk with protein extracted from plants. Using plant protein to replace part of animal protein can not only reduce the cost of cheese production, but also improve the nutritional value of cheese. In the cheese research and development field, the experiment of replacing part of animal protein with plant protein has become a new research and development direction [38].

Among them, mixed soybean cheese is the most studied double protein cheese. Soybean contains 35~40% protein, which is a high-quality source of plant protein. It has high nutritional value, contains various amino acids and unsaturated fatty acids necessary for the human body, and is also rich in minerals and vitamins. Soy protein plays an important role in the diet structure of many countries. The development of mixed soybean protein cheese can not only reduce the production cost of cheese, alleviate the shortage of milk source, but also promote the deep processing of soybean and increase the added value of soybean products.

Under the same ripening conditions, compared with ricotta cheese, mixed soybean cheese has larger pores and looser texture. Large particles of soy protein can reduce curd stability and affect the compactness of the casein structure [39]. Therefore, the current research on the quality of mixed cheese mainly focuses on the addition amount of soymilk, the processing method and the improvement of production technology.

**Figure 2:**
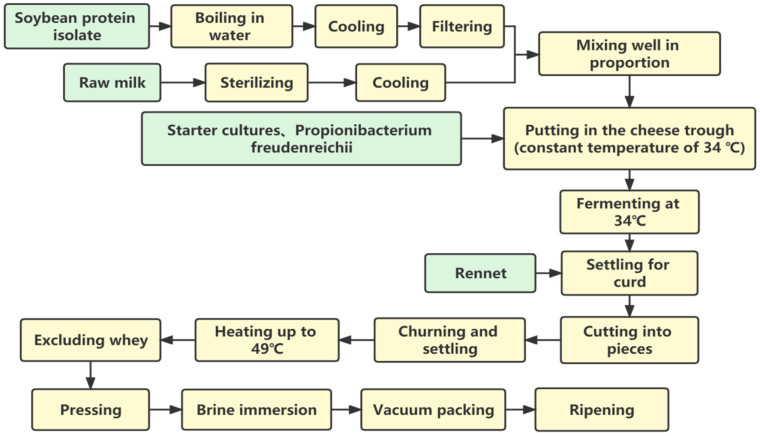
Process flow chart showing the production of mixed soybean cheese using soybean protein isolate and milk.

Yang [40] reported that when the content of soybean protein isolate was controlled at 4%, the muted taste of mixed cheese was greatly reduced, and the milky aroma was stronger. When the content of soy protein was more than 4%, the taste of milk cheese became rougher, the aroma of milk decreased, and the aroma of soy increased (Figure 2). Zhao et al. [41] found that the addition of soymilk resulted in a higher yield of Mozzarella mixed cheese and significantly reduced the fat content and firmness of the cheese samples. However, the addition of soymilk also made the cheese waterier, especially when the addition exceeded 10%. Its stretchability was significantly reduced, which was detrimental to its application on pizza. Bai et al. [42] found that with the increase in black soybean milk addition in the range of 2–6%, the water activity of cheese increased, the pH decreased, the hardness, elasticity, adhesiveness and chewiness increased. Based on the analysis of each index, the cheese made with black soybean milk 4% had a special flavor and proper indexes including color, texture, protein degradation and so on.

On the premise of not reducing the cheese yield, the addition of enzymatically hydrolyzed soymilk has a better effect on improving the texture of the mixed cheese. Li et al. [43] found that after adding soymilk and enzymatically hydrolyzed soymilk to cheddar cheese, the moisture content increased, and the fat content decreased significantly. As cheese matures, hardness and cohesion increased, while elasticity decreased. However, the cheese made by adding soymilk had poor shape and brittleness. Adding enzymolyzed soymilk could improve this phenomenon, and the protein structure formed by adding enzymolyzed soymilk was more compact. Han et al. [44] used 0.3% papain to hydrolyze soybean milk for 15 min before processing, which could significantly reduce the particle size of soybeans, reduce product hardness and smear work. Adding complex emulsified salt (sodium citrate: sodium tripolyphosphate: sodium hexametaphosphate) to the spread-type mixed soybean cheese could significantly improve the fineness and stability of the product.

Some researchers had pointed out that an important reason why consumers do not accept blended cheeses containing soymilk was its soy flavor [45]. At present, there are three main methods to remove the beany smell. The first is to discover and cultivate new soybean varieties through the improvement of raw materials; the second is to reduce the beany smell during processing by inactivating or inactivating the activity of lipoxygenase in soybeans; the third is to improve storage conditions [46]. Ali et al. [47] used protease and peptidase to produce flavored enzyme-modified cheese. The results showed that after enzymatic hydrolysis, the contents of amino acids, free fatty acids and volatiles in cheese were significantly increased, and the sensory properties were significantly improved. Han et al. [44] found that compared with ordinary refining, the soymilk obtained by anaerobic refining had lower overall volatile flavor substances, especially beany flavor substances. The types and contents of beany flavor components in the spread-type soybean cheese prepared by anaerobic refining were significantly reduced, and the sensory evaluation was higher.

At present, in addition to soy cheese, other plant-based mixed cheeses are also emerging. Shi et al. [48] developed a hazelnut processed cheese with an optimal dosage of 30% hazelnut, and the emulsifier included 1.2% sodium citrate and 1.2% compound phosphate. The prepared processed cheese had a sweet taste, fine texture and mellow hazelnut aroma. Wu [49] reported that almond pulp and milk were mixed at 45:55%, 5% starter was added for fermentation, 0.8% rennet and 0.06% CaCl2 were added for processing. The almond cheese with milky white color, smooth and uniform, moderate sour and sweet, and rich flavor could be obtained. Tian et al. [50] developed a fermented spread walnut cheese with walnut kernels as the main raw material. The additions of lipase and flavor protease were 0.2%, and the additions of whey protein, cream, and sucrose were 1.56%, 0.81%, and 6.37%, respectively. Walnut cheese was full of flavor, high nutritional value, and had better sensory qualities. Zhang [51] used red dates and skim milk as raw materials, added starter and rennet, and made red date cheese through curdling. The experiment found that adding 4% of jujube puree, the curd time was relatively short and the curd strength was the greatest. At this time, the cheese was rich in flavor, pure in frankincense, and of the best quality. Chen [52] invented a preparation method of whole grain cheese with mild flavor and tender taste. Purple potato, sweet potato, wheat, red bean and oat were milled to make multigrain juice, mixed with skim milk, maltose, fructo oligosaccharide and honey, and then inoculated with lactic acid bacteria to ferment the curd.

At present, the research on double protein cheese is very extensive and in-depth, and the scientific research results have been applied to actual production. The development of various forms of cheese is of great benefit to the cultivation of the cheese market. The growing double protein cheese has great development potential and wide application prospects in the dairy industry.

### 2.4. Calf Double-Protein Milk Replacer

In order to wean the calves early, the calves should be fed with milk replacer (also known as artificial milk) instead of regular milk about 10 days after birth. At present, the use of milk replacer to cultivate and implement the early weaning technology of sucking calves has become a common technical means in the world dairy farming industry. Milk replacer raw materials are mainly composed of dairy by-products such as skim milk, whey protein concentrate, dry whey, etc. [53]. With the deepening of research and the development of milk replacer processing technology, low-cost and high-quality plant protein has become the main research direction for the development of milk replacer protein sources. High-quality plant protein and high proportion of milk protein have obvious effects on preventing and reducing calf diarrhea, and also on increasing daily weight gain of calves. Good economic benefits have been achieved by saving feeding costs [54].

The most widely used plant protein in calf milk replacer is soybean protein, wheat protein, rice protein, etc. Plant protein sources are rich and high in crude protein. The crude protein content of feed-grade soybean protein isolate, wheat hydrolyzed protein and rice protein provided in China can reach about 90%, 85% and 65%, respectively. Different sources of plant protein have different nutritional characteristics because of their different protein components and amino acid compositions and have different effects on the growth function of sucking calves [55]. Although plant protein has a slightly poorer amino acid balance, certain functional amino acids are abundant in plant protein. For example, wheat protein is rich in glutelin, accounting for 30% of the total amino acid [56]. In addition to synthesizing proteins to meet the needs of animal growth and maintenance, these functional amino acids are also necessary for the synthesis of various biologically active substances.

In the past studies, plant protein mainly had adverse factors such as poor solubility, low digestibility, poor amino acid balance and containing anti-nutritional factors [57]. However, with the development of science and technology, it is now possible to remove anti-nutritional factors through modification and processing, add different plant proteins to achieve amino acid balance, and add some enzymes or probiotics to improve the digestibility of plant proteins in animals (Table 1). Therefore, after using soybean protein isolate, wheat hydrolyzed protein and rice protein isolate as the protein source of milk replacer to partially replace milk-derived protein to feed calves, it can achieve a feeding effect similar to that of milk-derived protein [58].

The broad concept of “double protein” does not mean that there can only be one type of plant-derived protein added. In terms of human nutrition, the purpose of proposing the “double protein” project is to balance the diet, optimize the dietary structure, and improve the nutritional status. Therefore, adding two or more plant-derived proteins into milk replacer is particularly important for balancing amino acids and optimizing the dietary structure of livestock [71]. More and more studies have found that milk replacer composed of a variety of plant proteins have a combined effect, and the feeding effect is better than the combination of a single plant-derived protein and milk-derived protein.

Huang [72] reported that the feeding combination of 30% milk protein + 23.4% soybean protein concentrate + 23.3% rice protein isolate + 23.3% peanut protein concentrate was more in line with the nutritional needs of calves. For the same 30% milk protein retention, Liu et al. [73] found that the combined milk replacer with 40% soybean protein isolate, 10% wheat hydrolyzed protein and 20% rice protein isolate could give calves better growth performance. Raeth et al. [74] found that when 50% of milk-derived protein was replaced by soy protein isolate and wheat hydrolyzed protein in the same proportion, it would cause a decrease in daily weight gain and feed efficiency of calves. Studies had shown that high proportions of gliadin and glutelin in calf milk replacer can lead to reduced growth performance. However, as long as no less than 40% of retained colostrum was added or no less than 60% of whey protein was added, the normal growth and development of calves could be ensured.

Sucking calves are in the stage of rapid growth and development, and the level of energy intake directly affects the growth rate and nutrient metabolism of the body. It is very important to ensure an appropriate energy supply for sucking calves. Different protein sources of milk replacer have different energy utilization rates in animals. Compared with milk-derived protein, plant protein can reduce the metabolic rate of energy, nitrogen, calcium and phosphorus in calves. However, the effect of plant protein on the metabolic rate of energy, nitrogen, calcium and phosphorus in calves has a downward trend with the increase in age, and the adaptability of calves to plant protein is also continuously improved [72,75]. This is because plant protein contains a certain amount of fiber and rich nutrition, which has a significant promoting effect on the development of digestive organs such as the rumen and intestinal tract of the calf, and it lays a good foundation for the high production performance in the later period [76].

There were also differences in the effects of protein sources in milk replacers on the immune function of calves. Huang et al. [72] found that from the serum IgG, IgA, IgM and 1L-2 levels of calves, the stress caused by milk-derived protein, soybean protein and rice protein to calves was significantly lower than that of wheat protein and peanut protein. At the same time, compound plant protein could also increase the body’s deposition of nitrogen by increasing the levels of GH and IGF-1 in serum and improve the ability of tissue growth and development [73].

With the in-depth research on the development of plant protein, the development of milk replacer with plant protein as the protein source has a very good prospect. However, research on double-protein milk replacer is not sufficient, and other nutrients other than protein and amino acids have not been systematically studied. There is also a lack of relevant reports on the specific effects of feeding double-protein milk replacer on the microecology of the calf’s digestive tract and digestion and absorption function, as well as on the subsequent production performance. Therefore, more research is needed to reveal the nutritional potential of double-protein milk replacers, so as to provide more of a theoretical basis for precision feeding in the breeding stage of calves.

### 2.5. Other Double Protein Dairy Products

Cereal milk powder is more nutritious and functional than milk powder, and supplements the dietary fiber needed by the human body without changing the taste of milk powder. Cereal milk powder can be consumed in breakfast or other meals as a staple functional beverage. When drinking milk on an empty stomach for breakfast, the protein in the milk will be converted into sugar to release energy, resulting in a waste of protein. The grain-added milk can be eaten directly as a staple food, so that the protein can be absorbed well, and the convenience and staple food of dairy products can be realized. Wang [77] invented a method for preparing oat milk powder. After the oat flakes were extruded by a twin-screw extruder, milk powder, xylitol and fructo oligosaccharide were added in a certain proportion to make oat milk powder. Zhang [78] invented a preparation method of corn milk powder. The corn flour was puffed and then pulverized, and 30% of the puffed corn flour, 68% of the milk powder, 1.8% of the sugar and 0.2% of the edible essence were mixed in a mixer to make the corn milk powder.

According to the growth and development characteristics of infants and young children, timely and reasonable addition of complementary foods plays an important role in promoting the healthy development of infants and young children. Rice protein is a recognized hypoallergenic protein, and various clinical studies have also shown that rice protein can be used as a hypoallergenic protein resource, especially suitable for infant food ingredients. Liu et al. [79] obtained a series of formulas through orthogonal experiments based on the nutritional characteristics of infants and young children in different periods. Taking 100 g as the standard, the nutritional rice flour for infants and young children in 0–6 months contained 46 g of rice flour, 41 g of first-stage milk powder, 7.2 g of vegetable and fruit powder, 3.6 g of FOS, and 2 g of various trace elements. Nutritional rice flour for infants aged 6–12 months contained 46 g of rice flour, 32 g of second-stage milk powder, 12.6 g of multigrain flour, 6.3 g of FOS, and 3 g of various trace elements.

Ice cream is a popular dairy product for summer, and the production of new, safe and healthy ice cream has become an industry trend. Wu et al. [80] used flaxseed meal as raw material, extracted flax protein with enzyme preparation, added flax protein content of 3%, skim milk powder content of 13%, cream content of 15% and sucrose content of 16%. The finished product was light brown, with pure fragrance and fine texture. It could be seen that adding flax protein can improve the quality of ice cream. Zheng et al. [81] used USPI-PLW to replace part of the milk powder. Compared with ordinary low-fat ice cream, the expansion rate of ice cream was increased by 94.84%, the melting rate of ice cream was reduced by 26.86%. The product was in a stable condition, with a good appearance and smooth taste, which made its flavor more popular.

## 3. Perspectives for the Future

Based on the current research and development status of double-protein dairy products, several suggestions are put forward on the research and development ideas of this category of products in China in the future, which should be followed as below:

1. Increase the construction of high-quality beans and grain raw material bases to produce high-quality plant protein raw materials. On the premise of not destroying the taste, try to ensure the integrity and availability of raw materials, avoid waste of resources, and improve the nutritional quality of products.
2. Make full use of compound biological enzymatic hydrolysis preparation technology and probiotic fermentation technology. Accelerate the screening and development of probiotic strains, starters and probiotic preparations suitable for various plant-based fermentations, create natural and green manufacturing technologies, and improve the flavor and nutritional quality of products.
3. Strengthen research on product stability, and continuously improve related key production technologies and equipment, such as starch modification technology, pulsed electric field, ohmic heating, high- and ultra-high-pressure homogenization. Research and develop new production technology and equipment and adopt more advanced cold sterilization and aseptic packaging methods to improve product stability and extend shelf life.
4. Appropriately increase plant-based raw materials such as vegetables and Chinese herbal medicines with the same origin of medicine and food to enrich product types. In particular, new products with special flavor and nutritional and health characteristics can be purposefully developed for different consumer groups. For infants and young children, fruit and vegetable raw materials rich in vitamins and minerals can be developed and strengthened; for middle-aged and elderly people, some medicinal and food homologous ingredients rich in antioxidants and anti-aging substances can be added.

## 4. Conclusions

Concerns about environmental impact and sustainability, animal welfare and personal health issues have fueled consumer demand for plant protein. However, the transition towards greener diets is being hampered by the poor acceptance of vegan foodstuffs among consumers. Mixed animal/plant products to familiar dairy products offer a new field of innovation. Therefore, plant-based proteins were used in a variety of dairy products. This comprehensive review presents the research and application of plant protein in the dairy industry, a distinctive and interesting topic for researchers in food technology, nutrition and dietetics. The continuous development of new blended products and the expansion of the application of plant protein in dairy products can promote a greater role of plant protein in human society. The current focus is on possible ways to improve nutritional properties through processing, such as the use of enzymes, the selection of raw materials based on their protein quality, advanced processing and technological interventions. There is also a need to ensure the palatability and acceptability of double-protein dairy for the population.

## Figures and Tables

**Table 1:** Main functional properties of plant protein components.

Classification	Main Source	Advantages	Disadvantage	Technical Transformation	References
Globulin	Soybean	− Balanced composition of amino acid. − Good solubility.	− High content of antigen protein, which affects the intestinal health of young animals.	− Modification to inactivate antigenic proteins.	[59,60,61]
Gliadin	Corn, Wheat, Sorghum	− High content of glutamine, which can repair the intestinal mucosa of young animals.	− The increase in feed viscosity with the increase in gliadin content. − Not easily digested by animal endogenous digestive enzymes.	− The control of addition amount. − Directed enzyme digestion or microbial fermentation.	[62,63,64]
Glutelin	Rice	− Balanced composition of amino acid. − Easily digested by animal endogenous digestive enzymes.	− Low solubility in aqueous solution.	− Modification	[65,66,67]
Albumin	Corn, Wheat	− Balanced composition of amino acid.	− Existence of trypsin inhibitors and allergens.	− Modification − Gene modification	[68,69,70]

## Data Availability

Not applicable.
